# Multibody-Based Input and State Observers Using Adaptive Extended Kalman Filter

**DOI:** 10.3390/s21155241

**Published:** 2021-08-03

**Authors:** Antonio J. Rodríguez, Emilio Sanjurjo, Roland Pastorino, Miguel Á. Naya

**Affiliations:** 1 Laboratorio de Ingeniería Mecánica, University of A Coruna, Escuela Politécnica Superior, Mendizábal s/n, 15403 Ferrol, Spain; emilio.sanjurjo@udc.es (E.S.); miguel.naya@udc.es (M.Á.N.); 2 Test Division, Siemens Digital Industries Software, Interleuvenlaan 68, B-3001 Leuven, Belgium; roland.pastorino@siemens.com

**Keywords:** adaptive Kalman filter, multibody dynamics, nonlinear models, virtual sensing, multibody based observers

## Abstract

The aim of this work is to explore the suitability of adaptive methods for state estimators based on multibody dynamics, which present severe non-linearities. The performance of a Kalman filter relies on the knowledge of the noise covariance matrices, which are difficult to obtain. This challenge can be overcome by the use of adaptive techniques. Based on an error-extended Kalman filter with force estimation (errorEKF-FE), the adaptive method known as maximum likelihood is adjusted to fulfill the multibody requirements. This new filter is called adaptive error-extended Kalman filter (AerrorEKF-FE). In order to present a general approach, the method is tested on two different mechanisms in a simulation environment. In addition, different sensor configurations are also studied. Results show that, in spite of the maneuver conditions and initial statistics, the AerrorEKF-FE provides estimations with accuracy and robustness. The AerrorEKF-FE proves that adaptive techniques can be applied to multibody-based state estimators, increasing, therefore, their fields of application.

## 1. Introduction

In many applications, it is required to acquire data in order to analyze the performance of a particular machine, to make decisions through control strategies or to gather data for durability purposes. Most of the time, it is possible to get all the data through sensors. However, there are particular cases where a sensor cannot be installed due to economical or technical issues. In these situations, data acquisition relies on estimations.

Kalman filter [1] stands out as one of the most used estimation techniques. It combines a model describing the system with sensor measurements. The estimations are based on the statistical properties of the desired variables conditioned on the sensors measurements [2]. Although the Kalman filter was designed for linear systems, several approaches have been developed in the literature extending its use to non-linear models. Such is the case of the extended Kalman filter (EKF) or unscented Kalman filter (UKF) [2]. These approaches introduced the Kalman filter to real problems, which are predominantly non-linear.

Similarly, the interest for mulibody models has grown in the recent years in several fields of the industry, such as automotive, biomechanics or machinery. Through multibody dynamics, a system can be modeled with high accuracy [3], at expenses of a moderate computational cost. Recently, multibody models have started to be used in combination with Kalman filters for estimation. Such is the case of virtual sensing in automotive applications [4]. However, the assembly between a multibody model and a Kalman filter is not trivial due to the high non-linearities of multibody models.

The first approach presented in the literature is [5], where an EKF was combined with a simple mechanism. However, the complexity of the implementation and the computational cost limits its application. Several approaches have been presented later on [6,7,8,9]. In [7], an UKF based on a multibody model is proposed, reducing the complexity of the implementation. However, it entails high computational cost: the estimations are based on a set of sample points propagated through the model. Hence, it requires to simulate as many multibody models as sample points each time step. From the work presented in [8], a new Kalman filter known as error-state extended Kalman filter (errorEKF) shows accurate results while reducing the computational cost. In addition, the filter entails low coupling with the model. Thus, the implementation of any model becomes simpler. The errorEKF has been improved in more recent works, extending its estimations to inputs [9] and parameters [10].

The Kalman filter, besides being developed for linear systems, assumes that the noise of the measurements and the process (or system) are zero-mean white Gaussian with known covariance matrices [1]. These assumptions are hard to satisfy in real scenarios. The use of wrong statistics in the design of the filter can lead to large estimation errors or even a divergence in the filter [11]. In practice, these matrices are estimated through a tedious trial-and-error procedure. This is especially critical for the process noise covariance matrix (PNCM), since it is not possible to determine the accuracy of the model. Regarding the measurement noise covariance matrix (MNCM), each sensor is characterized and, hence, an initial value can be approximated.

However, even though a proper value of the noise covariance matrices is found, they remain constant reducing the robustness of the filter [10,12]. In some scenarios, the system can experiment a noticeable change during a particular maneuver. This change can affect the model accuracy and, thus, invalidate the initial noise covariances. This explains the increasing interest on developing methods for noise covariance matrices estimation [13]. These methods are usually known as Adaptive Kalman filters (AKF).

Adaptive Kalman filters can be divided in two major approaches: multiple-model based adaptive estimation (MMAE) and innovation-based adaptive estimation (IAE). Both approaches share the same concept of using the new information of the innovation sequence of the filter (difference between real and estimated measurements) [14]. MMAE methods implement a bank of several different models and compute the probability for each model to be the true system model given the measurement sequence and under the assumption that one of the models in the bank is the correct one. IAE performs the adaptation based directly on the innovation sequence, using the fact that the innovation sequence is white noise if the filter has the right values of the covariance matrices [15].

Multibody models can be computationally expensive and achieving real-time performance in practical applications is a challenge [16]. Hence, executing several models in parallel following the MMAE approach would compromise real-time performance and, thus, the viability of the solution.

Focusing on the IAE, there are many alternatives presented in the literature. In [14], an approach based on the maximum likelihood criteria (ML) is proposed to estimate both the PNCM and MNCM for inertial navigation, where Kalman filters are employed to overcome the lack of GPS signals in certain areas. The proposed approach is also compared against conventional Kalman filters. The ML aims to maximize the probability that a set of data (i.e., innovation) is observed for a given a parameter (i.e., noise covariance matrices). The ML depends on a window of past innovation data. If the sample data grows without bound, the estimate converges to the true value of the covariance matrices. As a counterpart, the ML could be biased for small sample sizes (over-trained). This issue is addressed in [17] by combining the ML with a fuzzy control in order to select only the viable satellites for the estimation, removing outlier data from the innovation sequence.

In [18], an IAE adaptive Kalman filter is also proposed for sensor fusion in INS/GPS navigation. The algorithm is equivalent to the maximum likelihood criteria. Combining the GPS with an IMU, the position can be accurately tracked, even in environments without GPS signal.

For spacecraft navigation, in [12], an AKF based on ML combined with fuzzy logic is proposed. The fuzzy logic is based on the discrepancy between the estimated covariance and the theoretical covariance bounds. The use of fuzzy logic helped to reduce the computational cost. However, the use of fuzzy logic incremented the estimation errors.

In [19], a Sage-Husa (SH) filter is combined with a Kalman filter to obtain the angular velocities of a vehicle from an inertial navigation system which uses only accelerometers. The SH algorithm is equivalent to those resulting from maximum likelihood estimation [20]. It is based on the maximum a posterior probability (MAP), which maximizes the probability of a parameter for a given set of data. It means that the likelihood is weighted by the prior information. In practice, it can be seen as applying weights in the ML estimates, giving more relevance to the most recent estimations. Sage-Husa algorithm is an alternative to the ML when there are not enough data available. However, it cannot estimate the PNCM and MNCM simultaneously without causing divergence in the estimations [21].

The SH algorithm is also used with modifications in [22] to estimate the MNCM of an UKF for state estimation in a vehicle. A similar approach is followed in [21], where a robust UKF is complemented with the SH algorithm for estimating the PNCM for autonomous underwater vehicle acoustic navigation.

Another approach for adaptive Kalman filtering is known as Variational Bayesian (VB). While ML and MAP give a fixed value for the noise covariance matrices (point estimators), the VB gives a probability density function. The advantage of this method is that it mitigates the effect of over-trained filters [23]. As an example, the VB is applied in [24] in order to improve the measurements of inertial navigation systems. It shows robustness against anomalous measurements. As a counterpart, the VB assumes that the PNCM is accurately known. Thus, it can be only used for estimating the states and the MNCM [25]. This is an important drawback since, as reflected in [26,27], the filter performance will decrease with the uncertainties of the PNCM. The VB approach is extended in [26,28] including the calculation of the predicted error covariance matrix instead of the process covariance matrix seeking for higher accuracy. In the same way, in [29], the VB approach is enhanced by its combination with a Sage-Husa algorithm for the PNCM estimation achieving satisfactory results.

The aim of this work is to explore the use of adaptive algorithms with multibody based Kalman filters, solving the actual limitation of the unknown statistics of the noise covariance matrices. For that purpose, a Kalman filter based on multibody models has to be combined with an adaptive method. This implies to study how to combine the different equations involved, performing the required modifications. Most of the research made in adaptive Kalman filtering is related to navigation applications. However, multibody models present strong non-linearities that can affect the performance of the adaptive filters. To the authors’ knowledge, adaptive Kalman filtering is a technique that has never been applied to multibody-based estimation and, at the state of the art of Kalman filtering in this field, it is the next natural step to be taken.

As novelty, this work presents a state and input estimator for multibody models, which includes an adaptive methodology in order to estimate the noise covariance matrices. The lack of knowledge on the statistics of the system is an actual limitation for industrial applications of multibody-based estimation. That is the case, for example, of automotive applications, where the maneuvers are continuously changing and adjusting the noise covariance matrices following a trial-and-error procedure is not viable. Following the presented approach, this limitation can be overcome, and the use of estimation based on multibody models can be increased in real industrial applications.

## 2. Materials and Methods

This work continues the development of an accurate and efficient Kalman filter for multibody models. Starting from the work presented in [9], the errorEKF with force estimation is extended with adaptive methods improving its accuracy and robustness. The errorEKF with force estimation is one of the best options in terms of accuracy and computational efficiency for multibody-based Kalman filtering. Regarding the adaptive method, whereas the Variational Bayesian can only be used for estimating the measurement noise covariance matrix, the Sage-Husa and maximum likelihood can estimate the process and measurement noise covariance matrices. The former has shown lack of stability if both matrices are estimated simultaneously. Hence, the filter of this work combines the maximum likelihood method with the errorEKF with force estimation.

**Figure 1:**
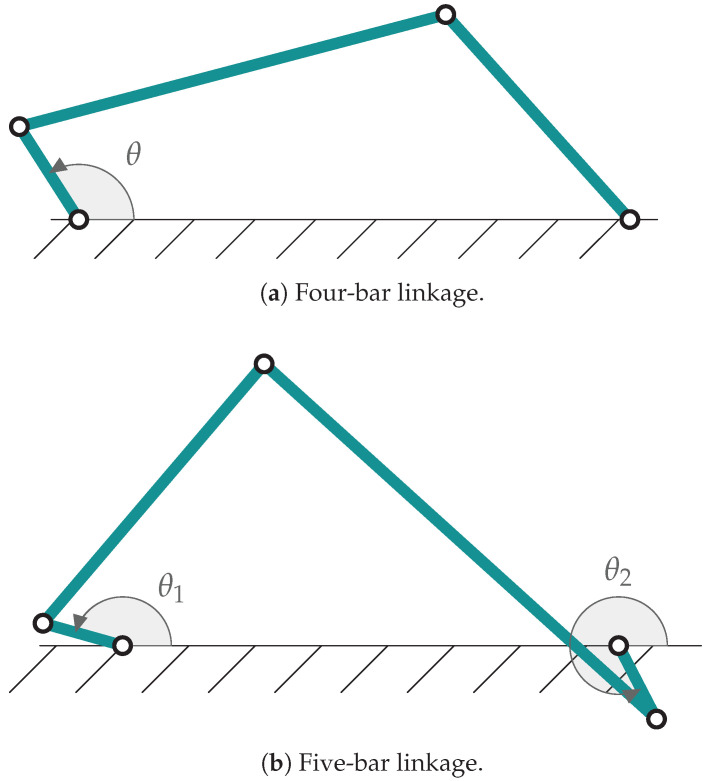
Mechanisms employed in this work.

The adaptive techniques used in this work are evaluated when applied to mechanisms described by multibody models. In order to present a fair comparison with the previous version of the filter, the mechanisms employed in this work are the same as in [9]: a four bar linkage (Figure 1a) and a five bar linkage (Figure 1b). The dimensions of both mechanisms are summarized in Table 1 and Table 2.

### 2.1. Multibody Modeling

The input and state observer that is used in this work, the errorEKF with force estimation, can be applied to any multibody formulation and integrator [8]. This is one of its main advantages, since it reduces the complexity of implementing estimators based on multibody models (MBS). For example, in [9], an augmented Lagrangian formulation of index 3 (ALI3P) using natural coordinates is employed. Meanwhile, in [10], the multibody model is based on a semi-recursive method using relative coordinates.

In this work, the ALI3P formulation is selected following the work in [9]. First presented in [30], this formulation is widely used in multibody applications due to its efficiency and robustness [16,31,32,33,34]. The formulation is briefly described next. The reader is referred to [30,31,35] for further details. The multibody models are simulated using MBDE, a MATLAB${}^{®}$ Open Source toolbox (See https://github.com/MBDS/mbde-matlab (accessed on 12 May 2021)).

The dynamics of a multibody system can be described by an index-3 augmented Lagrangian formulation as,

(1) $$M\overset{..}{q}+{\Phi_{q}}^{T}\alpha \Phi +{\Phi_{q}}^{T}\lambda^{*}=Q$$

being M the mass matrix, $\overset{..}{q}$ the accelerations of the natural coordinates, Φ the constraints vector, $\Phi_{q}$ the Jacobian matrix of the constraint equations with respect to the generalized coordinates q, α the penalty factor, Q the vector of generalized forces, and $\lambda^{*}$ the vector of Lagrange multipliers obtained through the following iteration process [35],

(2) $$\lambda_{i+1}^{*}=\lambda_{i}^{*}+\alpha \Phi_{i+1},\;\;i=0,1,2\ldots$$

where *i* is the index of the iteration.

It should be noted that this method directly includes the constraint equations at position level as a dynamical system, penalized by a large factor, α. The larger the penalty factor, the better the constraints will be achieved. In order to avoid numerical ill conditioning, a factor between $10^{7}$ and $10^{9}$ (depending on the mass of the system) gives excellent results for double arithmetic [3].

In order to integrate Equation (1), the implicit single-step trapezoidal rule has been adopted. The corresponding difference equations in velocities and accelerations can be derived as,

(3) $$\begin{array}{c} {\dot{q}}_{n+1}=\frac{2}{\Delta t}q_{n+1}+{\dot{q}}_{n}^{*};\;\;{\dot{q}}_{n}^{*}=-\left(\frac{2}{\Delta t}q_{n}+{\dot{q}}_{n}\right) \end{array}$$

(4) $$\begin{array}{c} {\overset{..}{q}}_{n+1}=\frac{4}{\Delta t^{2}}q_{n+1}+{\hat{\overset{..}{q}}}_{n}^{*};\;\;{\hat{\overset{..}{q}}}_{n}^{*}=-\left(\frac{4}{\Delta t^{2}}q_{n}+\frac{4}{\Delta t}{\dot{q}}_{n}+{\overset{..}{q}}_{n}\right) \end{array}$$

where Δt is the time-step. Introducing the previous equations in Equation (1) leads to a nonlinear system of equations, f, in which the positions at time-step n+1 are the unknowns. Hence,

(5) $$f(q_{n+1})=0$$

Since Equation (5) is a nonlinear system of algebraic equations, the Newton–Raphson iteration can be used to find a solution. The residual vector is,

(6) $$f\left(q\right)=\frac{\Delta t^{2}}{4}\left(M\overset{..}{q}+{\Phi_{q}}^{T}\alpha \Phi +{\Phi_{q}}^{T}\lambda^{*}-Q\right)$$

and the approximated tangent matrix,

(7) $$\frac{\partial f\left(q\right)}{\partial q}=M+\frac{\Delta t}{2}C+\frac{\Delta t^{2}}{4}\left({\Phi_{q}}^{T}\alpha \Phi_{q}+\bar{K}\right)$$

where $\bar{K}$ and C represent the contribution of damping and elastic forces of the system.

After converging to a solution, the positions satisfy the equations of motion and the constraint conditions, Φ=0. However, there is no guarantee on satisfying the constraint equations at velocity, $\dot{\Phi }=0$, and acceleration level, $\overset{..}{\Phi }=0$, since they were not imposed. To overcome this issue, the velocities and accelerations are projected,

(8) $$\frac{\partial f(q)}{\partial q}\dot{q}=\left[M+\frac{\Delta t}{2}C+\frac{\Delta t^{2}}{4}\bar{K}\right]\tilde{\dot{q}}-\frac{\Delta t^{2}}{4}{\Phi_{q}}^{T}\alpha \Phi_{t}$$

(9) $$\frac{\partial f\left(q\right)}{\partial q}\overset{..}{q}=\left[M+\frac{\Delta t}{2}C+\frac{\Delta t^{2}}{4}\bar{K}\right]\tilde{\overset{..}{q}}-\frac{\Delta t^{2}}{4}{\Phi_{q}}^{T}\alpha \left({\dot{\Phi }}_{q}\dot{q}+{\dot{\Phi }}_{t}\right)$$

where $\tilde{\dot{q}}$ and $\tilde{\overset{..}{q}}$ are the velocities and accelerations obtained from the integration process, i.e., non-compliant with the constraints equations.

### 2.2. Adaptive Error-State Extended Kalman Filter with Force Estimation (AerrorEKF-FE)

The errorEKF with force estimation is based on indirect estimation, also known as error-state estimation. It combines the efficiency of an EKF filter with the easy integration of the dynamical system in the UKF filters [36]. The coupling between the filter and model is limited to share information regarding the states, which are the error in the kinematics of the model. The multibody model can be used without modifications and, thus, any formulation or integrator can be employed.

**Figure 2:**
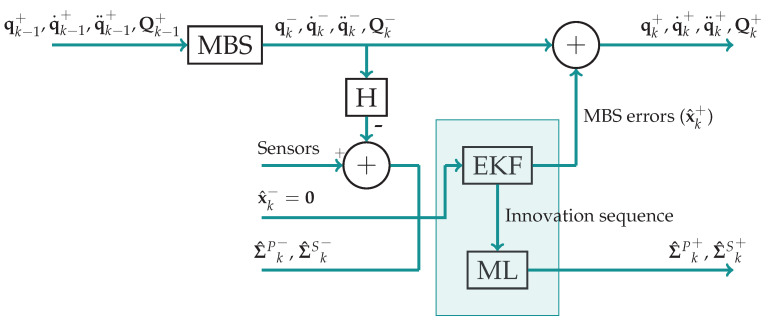
Simplified flow diagram of a time step of the adaptive errorEKF with force estimation. MBS refers to the multibody system, whose output is the predicted position, velocities and accelerations of the system. H is the observation matrix, employed to obtain the *virtual* measurements, which are compared with the measurements from the sensors. ${\hat{\Sigma }}^{P}$ and ${\hat{\Sigma }}^{S}$ are the PNCM and MNCM, respectively, where the superscripts ${}^{-}$ and ${}^{+}$ refer to the a priori and *a posteriori* estimations. EKF represents the application of the extended Kalman filter equations, whose outputs are the estimated errors in the predicted state vector (MBS errors). ML refers to maximum likelihood and is where the noise covariance matrices are estimated.

The diagram of Figure 2 illustrates the procedure followed by the adaptive errorEKF with force estimation for a time step *k*. The method starts integrating the multibody equations, obtaining the predicted coordinates $q_{MBS}$, velocities ${\dot{q}}_{MBS}$ and accelerations ${\overset{..}{q}}_{MBS}$. These coordinates are later used to obtain the *virtual* measurements $h(q_{MBS},{\dot{q}}_{MBS},{\overset{..}{q}}_{MBS})$. Later, the equations of the filter are used to perform the estimation.

#### 2.2.1. State and Input Estimation

For estimating the states and inputs of the system, the state vector consists in the *error* in position, velocity and acceleration of the degrees of freedom of the mechanism, ensuring a certain level of independence within the states. Hence,

(10) $$x^{T}=\left[\Delta z^{T},\Delta {\dot{z}}^{T},\Delta {\overset{..}{z}}^{T}\right]$$

where Δz, $\Delta \dot{z}$, $\Delta \overset{..}{z}$ are the errors in position, velocity and acceleration of the independent coordinates (coincident with the degrees of freedom), respectively, such that,

(11) $$\begin{array}{c} z=z_{MBS}+\Delta z \end{array}$$

(12) $$\begin{array}{c} \dot{z}={\dot{z}}_{MBS}+\Delta \dot{z} \end{array}$$

(13) $$\begin{array}{c} \overset{..}{z}={\overset{..}{z}}_{MBS}+\Delta \overset{..}{z} \end{array}$$

being $z_{MBS}$, ${\dot{z}}_{MBS}$, ${\overset{..}{z}}_{MBS}$ the *virtual* positions, velocities and accelerations of the degrees of freedom (i.e., the coordinates predicted by the multibody model), and z, $\dot{z}$, $\overset{..}{z}$ the *real* positions, velocities and accelerations of the degrees of freedom of the mechanism. It should be noted that estimating the accelerations is closely related with the estimation of the forces, as can be seen from Equations (22)–(24).

Combining the information from the *real* and *virtual* measurements into the Kalman filter, the state of the multibody model is corrected. Thus, the expected error for the next time step is null. As a consequence, the propagation phase takes the form of,

(14) $$\begin{array}{c} {\hat{x}}_{k}^{-}=0 \end{array}$$

(15) $$\begin{array}{c} P_{k}^{-}={f_{x}}_{k-1}P_{k-1}^{+}{f_{x}}_{k-1}^{T}+{{\hat{\Sigma }}^{P}}_{k-1}^{+} \end{array}$$

where P is the covariance matrix of the estimation error, $f_{x}$ is the Jacobian matrix of the transition model and ${\hat{\Sigma }}^{P}$ is the estimated covariance matrix of the plant or process (i.e., the multibody model), referred as PNCM. Note that since the filter is adaptive, the value of ${\hat{\Sigma }}^{P}$ is estimated each time step. For the propagation phase, the best estimation of the ${\hat{\Sigma }}^{P}$ is the one obtained in the previous time step.

Equations (14) and (15) are equivalent to the equations employed in the discrete extended Kalman filter (DEKF) [2,37]. Following [9], the Jacobian matrix of the transition model can be derived through a forward Euler integrator of the states and a random walk for modeling the acceleration error,

(16) $$f_{x}=\left[\begin{array}{ccc} I+\frac{1}{2}\frac{\partial \Delta \overset{..}{z}}{\partial z}\Delta t^{2} & I\Delta t+\frac{1}{2}\frac{\partial \Delta \overset{..}{z}}{\partial \dot{z}}\Delta t^{2} & \frac{1}{2}I\Delta t^{2}\\ \frac{\partial \Delta \overset{..}{z}}{\partial z}\Delta t & I+\frac{\partial \Delta \overset{..}{z}}{\partial \dot{z}}\Delta t & I\Delta t\\ 0 & 0 & I \end{array}\right]$$

where the terms $\frac{\partial \Delta \overset{..}{z}}{\partial z}$ and $\frac{\partial \Delta \overset{..}{z}}{\partial \dot{z}}$ reflect the effect of the acceleration error through the force models of the multibody model. Deriving expressions for these terms is not straightforward and is out of the scope of this work. The reader is referred to [9,38] for a comprehensive explanation on how to obtain these terms.

Following the equations defined for the DEKF [2,37], the corrections can be propagated through the states obtaining the *a posteriori* state vector, ${\hat{x}}^{+}$, yielding,

(17) $$\begin{array}{cc} y_{k} & =o_{k}-h(q_{MBS},{\dot{q}}_{MBS},{\overset{..}{q}}_{MBS}) \end{array}$$

(18) $$\begin{array}{cc} \Sigma_{k} & ={h_{x}}_{k}P_{k}^{-}{h_{x}}_{k}^{T}+{{\hat{\Sigma }}^{S}}_{k-1}^{+} \end{array}$$

(19) $$\begin{array}{cc} K_{k} & =P_{k}^{-}{h_{x}}_{k}^{T}\Sigma_{k}^{-1} \end{array}$$

(20) $$\begin{array}{cc} {\hat{x}}_{k}^{+} & =0+K_{k}y_{k} \end{array}$$

(21) $$\begin{array}{cc} P_{k}^{+} & =\left(I-K_{k}{h_{x}}_{k}\right)P_{k}^{-} \end{array}$$

being $y_{k}$ the innovation sequence, $o_{k}$ the measurements, $\Sigma_{k}$ the innovation covariance matrix, $K_{k}$ the Kalman gain, and ${\hat{\Sigma }}^{S}$ the covariance matrix of the sensors or measurements (MNCM). Similarly to ${\hat{\Sigma }}^{P}$ in Equation (15), ${\hat{\Sigma }}^{S}$ can vary within time steps. Hence, ${{\hat{\Sigma }}^{S}}_{k-1}^{+}$ is the more accurate value for propagating the corrections. The state vector and covariance matrix are estimated in Equations (20) and (21), respectively.

From the *a posteriori* state vector, ${\hat{x}}^{+}$, and through Equation (13), the estimated position $\hat{z}$, velocities $\hat{\dot{z}}$ and accelerations $\hat{\overset{..}{z}}$ for the time step *k* are obtained. Since the model relies on the set of dependent coordinates, it is required to compute the errors in these coordinates. Projecting the independent coordinates over the constraints manifold, the dependent coordinates can be obtained as stated in [9].

After correcting the states of the model, the forces (or inputs) can be estimated. The error in forces can be seen as the forces required to avoid the error in accelerations, which is referred as ΔQ. Since (in general) there are more dependent coordinates than degrees of freedom, there are infinite force combinations producing the same effect. Hence, depending on the application, different criteria should be considered in order to find a proper force distribution [10]. A common solution, valid for simple mechanisms, is to assume that the unknown forces are applied to the degrees of freedom. Therefore,

(22) $$\begin{array}{c} \Delta Q_{k}^{q}=0 \end{array}$$

(23) $$\begin{array}{c} \Delta Q_{k}^{z}=\Delta Q_{k-1}^{z}+\bar{M}\Delta \hat{\overset{..}{z}} \end{array}$$

(24) $$\begin{array}{c} \Delta Q={\left[\begin{array}{cc} {\left(\Delta Q^{z}\right)}^{T} & {\left(\Delta Q^{q}\right)}^{T} \end{array}\right]}^{T} \end{array}$$

being $\Delta Q^{q}$ and $\Delta Q^{z}$ the force correction acting on the dependent and independent coordinates (equivalent to the degrees of freedom), respectively.

#### 2.2.2. Process and Measurement Covariance Matrices Estimation

As reflected in Section 1, knowing the covariance matrices of the process and measurements noises is critical for a proper behavior of the filter in terms of accuracy and robustness. For the particular case of multibody models, the PNCM is the most critical. The PNCM is completely unknown and can vary depending on the maneuver due to, for example, external perturbations which are not modeled. On the other hand, the MNCM can be approximated by the information provided by the sensor manufacturer. In addition, fluctuations of this noise is not expected within maneuvers. In order to address this issue, in this work, the previous errorEKF with force estimation is extended with adaptive strategies for the PNCM. The effect of a proper estimation of the MNCM is also addressed.

Following the work presented in [14], through the maximum likelihood technique, the PNCM and MNCM can be estimated. The ML has the attractive property of uniqueness and consistency of the solution. This ensures, in a probabilistic sense, a convergence to the true value of the covariance matrices. However, the ML relies on a sliding window of the innovation sequence, whose size directly affects its performance: small sample sizes would lead to biased estimations.

In addition, the solution proposed in [14] is developed for navigation purposes. It assumes that the filter transition matrix, $f_{x}$, and the design matrix, h, are time invariant. This is not true when using the errorEKF. Thus, the algorithm should be adapted for the use-case of this work.

The detailed procedure for estimating the PNCM and MNCM through the ML method is presented in [14]. Before presenting the method, it should be noted the difference between probability and likelihood. Probability refers to the chances of an event to happen based on the statistical distribution of some data. Likelihood corresponds to finding the distribution that fits best to a given set of data.

The ML estimation aims to solve the question of, given the observed data, which are the model parameters that maximize the likelihood of the observed data occurring. The likelihood function can be defined as L(α|Z), where α are the parameters and Z the observed data. In innovation-based adaptive Kalman filtering, the parameters are the noise covariance matrices, and the observed data corresponds to the innovation sequence. In plain words, the ML estimates the noise covariance matrices searching for the values which give the maximum probability of obtaining a particular innovation sequence.

In order to calculate the likelihood, it is expressed as the probability of observing a certain dataset under an unknown statistical distribution, that is P(Z|α). Hence, the target is to find the value of α, which maximizes P(Z|α). If the sets of observed data, *N*, are independent, the likelihood of observing the data can be expressed as the product of the probability of observing each data point individually yielding,

(25) $$L\left(\alpha |Z\right)=\prod_{j=1}^{N}P\left(Z_{j}|\alpha \right)$$

where α, for this particular case, corresponds to the elements of the noise covariance matrices, and Z are the innovations of the errorEKF with force estimation, y. Regarding $\prod_{j=1}^{N}\left(\cdot \right)$, it implies the product of the full set of innovations. However, storing the entire set of past data is not viable for online estimation. Thus, a slide window is defined so that the filter only processes a fixed number of past events.

Assuming that the innovation sequence is Gaussian distributed, the likelihood function can be expressed as,

(26) $$L\left(\alpha |y\right)=\prod_{j=1}^{N}\frac{1}{\sqrt{{\left(2\pi \right)}^{m}\left|\Sigma_{j}\right|}}\exp^{-\frac{1}{2}y_{j}^{T}\Sigma_{j}^{-1}y_{j}}$$

being y the innovation sequence, Σ the covariance matrix, *m* the number of measurements per time step, |·| the determinant operator, and exp the natural base.

The value of α maximizing the likelihood can be obtained by deriving the likelihood function (Equation (25)) with respect to α and setting it to zero. However, differentiate Equation (25) is difficult. As an alternative, Equation (25) is simplified by taking the natural logarithm of the equation yielding,

(27) $$L\left(\alpha |y\right)=-\frac{1}{2}\sum_{j=j_{0}}^{k}\left[ln|\Sigma_{j}|+y_{j}^{T}\Sigma_{j}^{-1}y_{j}+c_{j}\right]$$

where $c_{j}$ is a constant term independent of the adaptive parameters, α. Maximizing the previous equation is equivalent to minimizing its negative version. Neglecting the constant terms, the maximum likelihood condition can be derived,

(28) $$\sum_{j=j_{0}}^{k}ln\left|\Sigma_{j}\right|+\sum_{j=j_{0}}^{k}y_{j}^{T}\Sigma_{j}^{-1}y_{j}=min$$

where *k* is the time step in which the covariance matrix is estimated and *j* is the counter inside the estimating window. Equation (28) aims at finding the covariance matrix, which results in the smallest error norm, being complementary to the state estimation.

As stated before, the solution for Equation (28) can be constructed by taking its derivative with respect to the adaptation parameters and setting the results to zero. This yields,

(29) $$\sum_{j=j_{0}}^{k}\left[tr\left\{\Sigma_{j}^{-1}\frac{\partial \Sigma_{j}^{S}}{\partial \alpha_{k}}\right\}-y_{j}\Sigma_{j}^{-1}\frac{\partial \Sigma_{j}^{S}}{\partial \alpha_{k}}\Sigma_{j}^{-1}y_{j}^{T}\right]=0$$

In addition, using Equations (15) and (18), the covariance matrix, $\Sigma_{k}$, can be replaced by $\Sigma^{P}$ and $\Sigma^{S}$. After some manipulation [14], the equation that maximizes the likelihood function for the adaptation parameters can be expressed as,

(30) $$\sum_{j=j_{0}}^{k}tr\left\{\left[\Sigma_{j}^{-1}-\Sigma_{j}^{-1}y_{j}y_{j}^{T}\Sigma_{j}^{-1}\right]\left[\frac{\partial \Sigma_{j}^{S}}{\partial \alpha_{k}}+{h_{x}}_{j}\frac{\partial \Sigma_{j-1}^{P}}{\partial \alpha_{k}}{h_{x}}_{j}\right]\right\}=0$$

From Equation (30), expressions for $\Sigma^{P}$ and $\Sigma^{S}$ can be obtained independently. For deriving the expression of $\Sigma^{P}$, it is assumed that $\Sigma^{S}$ is known and independent of α (i.e., $\frac{\partial \Sigma_{j-1}^{P}}{\partial \alpha_{k}}=0$). Under this assumption, the adaptation relies on $\Sigma^{P}$. This means that the covariance noises of the process are the adaptive parameters (i.e., $\alpha =\sigma^{2}$). Considering $\Sigma^{P}$ as a diagonal matrix leads to

(31) $$\sum_{j=j_{0}}^{k}tr\left\{\left[\Sigma_{j}^{-1}-\Sigma_{j}^{-1}y_{j}y_{j}^{T}\Sigma_{j}^{-1}\right]\left[0+{h_{x}}_{j}I{h_{x}}_{j}\right]\right\}=0$$

which can be reduced to

(32) $$\sum_{j=j_{0}}^{k}{h_{x}}_{j}^{T}\left[\Sigma_{j}^{-1}-\Sigma_{j}^{-1}y_{j}y_{j}^{T}\Sigma_{j}^{-1}\right]{h_{x}}_{j}=0$$

Introducing now the expressions of the errorEKF, the covariance matrix Σ can be replaced by $\Sigma^{P}$, leading to

(33) $${{\hat{\Sigma }}^{P}}_{k}=\frac{1}{N}\sum_{j=j_{0}}^{k}\left[\Delta x_{j}\Delta x_{j}^{T}+P_{j}^{+}-{f_{x}}_{j}P_{j-1}^{+}{f_{x}}_{j}^{T}\right]$$

where $P_{j}^{+}-{f_{x}}_{j}P_{j-1}^{+}{f_{x}}_{j}^{T}$ covers the change in the covariances between time steps, and $\Delta x_{k}$ is the state correction sequence. Since the state vector in the errorEKF, defined in Equation (10), is the error in the variables, and the a priori state vector is null,

(34) $$\Delta x_{k}={\hat{x}}_{k}^{+}-0$$

The same procedure can be followed to estimate the measurement noise covariance matrix. Thus, assuming that $\Sigma^{P}$ is known and independent of α, and that $\Sigma^{S}$ is diagonal,

(35) $${{\hat{\Sigma }}^{S}}_{k}=\frac{1}{N}\sum_{j=j_{0}}^{k}\left[y_{j}y_{j}^{T}-{h_{x}}_{j}P_{j}^{-}{h_{x}}_{j}^{T}\right]$$

Note that both estimations (Equations (33) and (35)) are considering that the transition matrix, $f_{x}$, and observation matrix, $h_{x}$, are time-varying.

Furthermore, the shape of both matrices for the errorEKF with force estimation is known. The information of the matrices shape is useful to help the algorithm to reach the convergence. Thus, the estimated PNCM and MNCM are modified in order to adapt them to their theoretical shape. The MNCM is a diagonal matrix composed by the covariance of each sensor. The PNCM, according to [9], is

(36) $${\hat{\Sigma }}^{P}=\left[\begin{array}{ccc} \;0\; & \;0\; & 0\\ \;0\; & \;0\; & 0\\ \;0\; & \;0\; & diag\left({\hat{\sigma }}^{2}\right) \end{array}\right]$$

where $diag\left({\hat{\sigma }}^{2}\right)$ is a diagonal matrix containing the variance of all the components of the discrete plant noise at acceleration level.

A last consideration is that the covariance matrices must be semi-definite positive. With this algorithm, this cannot be guaranteed. Having a negative definite matrix would lead the filter to failure. To ensure the semi-definite positiveness of the matrices, the negative values are set to their absolute value [39].

### 2.3. Methods

All the experiments of this work are performed in a simulation environment in MATLAB${}^{®}$, using the Open Source toolbox MBDE (See https://github.com/MBDS/mbde-matlab (accessed on 12 May 2021)). The simulations are executed in a single core of a PC with an Intel Core i5 at 3.50 GHz and 8 Gb of RAM. Since there is not a physical prototype, three multibody models of each mechanism are employed in order to replicate a real scenario through simulation. All the simulations are launched with a time step of 5 milliseconds, that is a frequency of 200 Hz. They are detailed hereafter:

- *Real mechanism*: This model represents the real version of the mechanism, providing the *ground truth*. It is equivalent to the physical mechanism. The sensor measurements are obtained from this model. The sensor data is gathered from the kinematics of the *real mechanism*. Since the sensor data is obtained from a simulation, it is required to add white noise to the simulated measurements in order to represent the noise properties of real sensors.
- *Model*: This model represents the modeling of the *real mechanism*. The *model* can be affected by several uncertainties. Even though multibody modeling can represent with high accuracy a real mechanism, it is always subjected to modeling errors. While the geometry of a system can be accurately determined, the force models present a high level of uncertainty. Hence, this model is modified, creating a discrepancy between model and real mechanism. It should be mentioned that the errors in the mass or the mass distribution will lead to a similar accuracy level regarding kinematics magnitudes, since both force and mass errors result in acceleration errors. In addition, while the mass usually remains constant, force models are prone to change during a maneuver. Thus, it is of interest to test the estimator under errors in force models. The modeling error introduced is of $1\;m/s^{2}$ in gravity acceleration. In addition, the initial value of the crank angle has an offset of π/16rad.
- *Observer*: The estimations are computed based on this model. Regarding the modeling, it is the same as the *model*. The difference is that it is combined with the filter. Thus, its motion and dynamics are corrected with the information of the sensors installed on the *real mechanism*.

**Figure 3:**
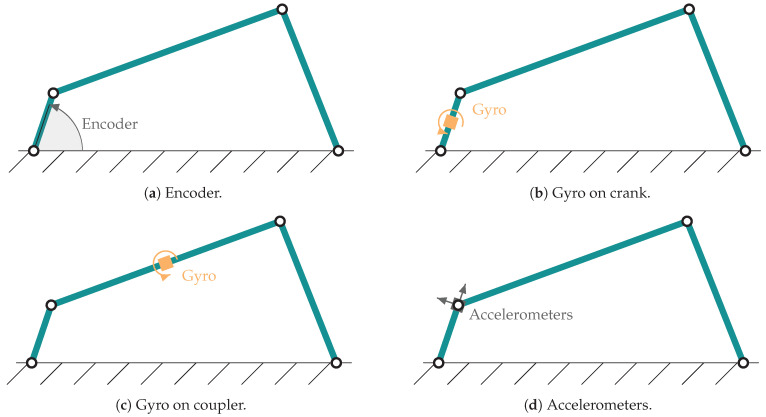
Sensor configurations considered in the four-bar linkage.

For the estimation, different sensors are considered. Although there are multiple possible configurations, for a reliable comparative with the results of [9], the same four combinations are considered. The four-bar and five-bar linkage share the same configurations for the sensors, duplicating the number of sensors in the case of the five-bar linkage, since it has two degrees of freedom. The sensor models are provided in [9]. The characteristics of each sensor are presented in Table 3. They are obtained from the technical data sheet of similar sensors. The four considered configurations are listed next:

- Encoder on the crank for measuring the crank angle, which is the degree of freedom (Figure 3a).
- Gyroscope on the crank for measuring the angular rates (Figure 3b).
- Gyroscope in the coupler of the mechanism (Figure 3c).
- Pair of accelerometers at the end of the crank, in such a manner that there is one sensitive axis parallel and another perpendicular to the crank (Figure 3d).

The performance of the proposed estimator is evaluated in terms of accuracy and robustness. For the accuracy, a set of tests is executed under different initial noise covariance matrices. It is expected that the filter converges to a similar accuracy level independently from the initial noise covariance matrices, due to the adaptive equations. With respect to the robustness, it is of interest that the estimator can cope with unexpected events during the simulations without causing a divergence in the estimations.

The numerical results of each test are analyzed using two different metrics: the root-mean square error (RMSE) and the confidence interval of the estimation errors. The RMSE is used to measure the accuracy of the estimations in position, velocity, acceleration and force acting in the degree of freedom of each mechanism. The RMSE can be obtained following Equation (37).

(37) $$RMSE=\sqrt{\frac{\sum_{i=0}^{N}{\left(Virtual_{i}-Real_{i}\right)}^{2}}{N}}$$

being *N* the number of samples gathered during the simulation. Virtual refers to the estimated value of the variable and Real to the reference value obtained from the *real mechanism*.

The confidence intervals of the errors give a different insight with respect to the RMSE on the accuracy of the estimator. For being reliable, the error of the estimations must be inside the confidence interval. Thus, the confidence interval of the estimation errors in position, velocity and acceleration are also provided. A confidence interval is calculated as a function of the standard deviation of a variable, which is related to the diagonal elements of the covariance matrix, P (Equation (21)). In this work, a confidence interval of 95% is selected. It can be calculated through Equation (38).

(38) $$CI=\bar{E}\pm 1.96\sqrt{\sigma_{i}}$$

where $\bar{E}$ is the mean estimation error of the *i*th variable and $\sqrt{\sigma_{i}}$ the standard deviation of the *i*th variable.

## 3. Results

To asses the performance of the proposed adaptive Kalman filter, different tests are launched. During the first test, the mechanisms are in the vertical plane and they are only affected by the gravity force. A batch of tests is executed under these conditions for different initial values of the PNCM. The results of each simulation are compared against the results obtained from the conventional errorEKF with force estimation. In a second test, the mechanisms are modified with the addition of a torsional spring in the crank. During the simulation, the spring is removed from the *real mechanism*, simulating a failure on a real machine. The AerrorEKF-FE is expected to overcome this new modeling error (together with the error in the gravity acceleration and initial position) in the *observer* and adapt the covariance matrices to the new situation.

The simulations are executed with a time step of 5 milliseconds, that is a frequency of 200 Hz. The measurements are gathered at 200 Hz. Hence, there is one measurement available at each time step. The errors introduced in the model for each test are of $1\;m/s^{2}$ in gravity acceleration and of an offset in the initial value of the crank angle π/16rad. In addition, for testing the robustness, a torsional spring is introduced in the crank. During the simulation, this spring is removed only from the *real mechanism*. The *observer* should detect this event through the sensor measurements and perform the required corrections.

### 3.1. Accuracy Test

For testing the convergence of the adaptive filter, different initial values of the PNCM are selected. While the MNCM can be approximated by testing the sensors, an initial value for the process noise is more difficult to obtain. Due to this inconvenience, it is of interest to test the robustness of the proposed filter under different initial values of the PNCM. The results are compared with the ones obtained from the errorEKF-FE. This would show the error reduction when using the AerrorEKF-FE if the initial PNCM is not close to the true value.

Following Equation (36), for the four-bar linkage, only one value is required. For the five-bar linkage, since it has two degrees of freedom, two elements of the PNCM matrix should be instantiated. For simplicity, both elements are set to the same value. The different initial values are summarized in Table 4.

Note that the initial values of both noise covariance matrices are used for the errorEKF-FE and AerrorEKF-FE. For the errorEKF-FE, these values are constant during the simulation, since it assumes that the PNCM is constant. Meanwhile, for the AerrorEKF-FE, these are the initial values of the PNCM, which are modified by the adaptive method. Hence, a total of six simulations for each sensor configuration are launched for each filter in order to compare their performance.

**Figure 4:**
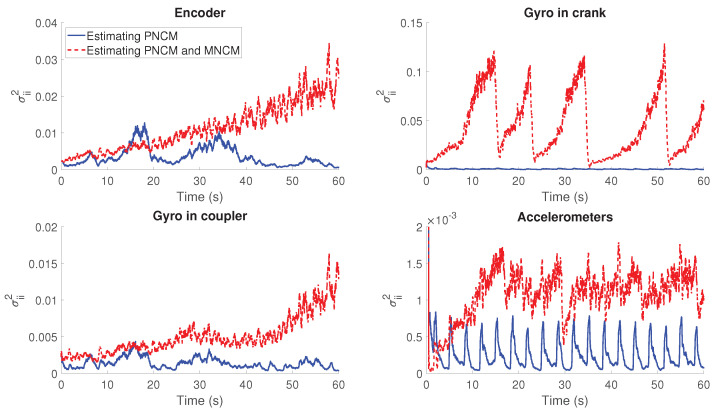
Estimated PNCM element in the four-bar linkage test for the case of estimating PNCM and MNCM simultaneously and estimating only the PNCM.

From the initial tests, an undesired behavior is detected. If both the PNCM and MNCM are estimated at the same time, the filter shows a trend to diverge. In Figure 4, the evolution of the estimated PNCM in the four-bar linkage is represented and compared with its evolution when the MNCM is not included in the estimation. Except for the case of the accelerometers, there is a trend to a continuous growth of the PNCM. This implies that the filter is reducing its trust on the model in exchange of the measurements.

The different behavior within configuration relies on the nature of the sensors. In the scenarios without accelerometers, there is a trend of divergence in the acceleration error. In addition, when using only an encoder, both the velocity and acceleration error diverge. Once that the estimator detects that the error is increasing through the innovation sequence, it starts to reduce the confidence in the model and trusting more in the sensors. However, correcting the acceleration error from measurements in position or velocity is not accurate. Hence, the error keeps growing causing the filter to diverge. At some point, this issue will lead to a failure of the filter. This, together with the non-linearities of the mechanisms, can result in unpredictable behaviors as the case of the gyroscope in the crank. Hence, for the rest of the experiments, only the PNCM is estimated.

**Figure 5:**
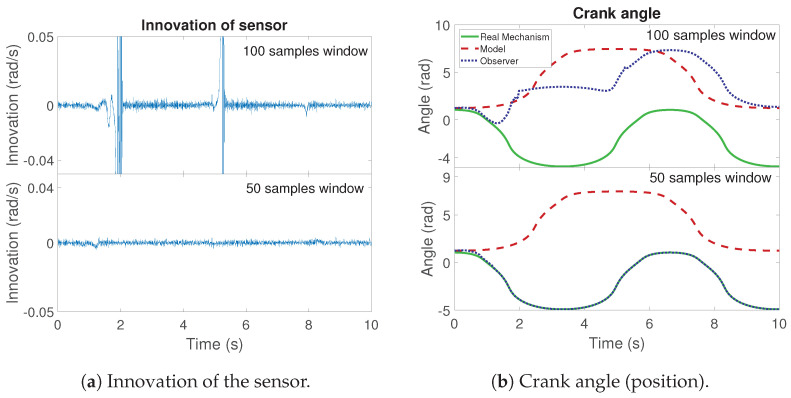
Comparison of innovation and crank angle for the simulation of the four-bar linkage with a gyroscope on the coupler for different window lengths.

Regarding the size of the sliding window for the innovation sequence, it should be noted that a large window leads to low mean errors in smooth maneuvers. However, a large window also implies slow corrections to the covariance matrix. Hence, in some tests, the delay in the correction can result in an error which is not corrected. This is the case shown in Figure 5. It corresponds to a simulation of the four-bar linkage with a gyroscope on the coupler and an initial process covariance noise of $\sigma_{ii,0}^{2}=10$, and a window length of 100 time steps. The same maneuver is performed with a window length of 50 samples. The innovation sequences are shown in Figure 5a. The value of the crank angle for each slide window size is shown in Figure 5b.

As can be seen in Figure 5a, with a window length of 100 samples, there are a notable difference between the estimated and measured angular rate of the coupler at particular moments of the maneuvers. In these points of the maneuver, the mechanism reaches a position where the crank and the rocker are parallel. In this situation, the possible velocities of the coupler are limited. Thus, for an error in position, the error in velocity can be incorrigible. This is a consequence of the non-linearity of the system.

Comparing the results between different window sizes, it is possible to appreciate the effects of the samples considered for the estimation. With a large window, the filter cannot correct with immediacy the error. Although it is able to correct the error in terms of crank angular rate, as is shown in the innovation sequence, a permanent offset appears in the position of the crank angle. Meanwhile, with a short window length, the influence of previous values is reduced and the filter can perform the correction properly. Hence, the tests of this work are executed with a window of 50 samples for the innovation sequence.

There is no a general criteria for selecting the length of this window. It should be adjusted depending on the nature of the maneuver aiming to include only the relevant information of past events. The main idea is to avoid the effect of past events which do not have relation with the actual state of the mechanism. If a maneuver is close to steady state, a large window can be selected. On the other hand, a short window length is adequate in cases where the system changes.

**Figure 6:**
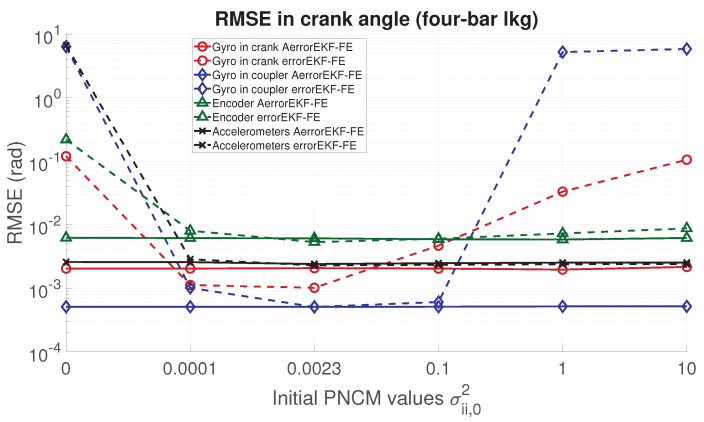
RMSE in the crank angle (position) provided by the observers for the four-bar linkage.

**Figure 7:**
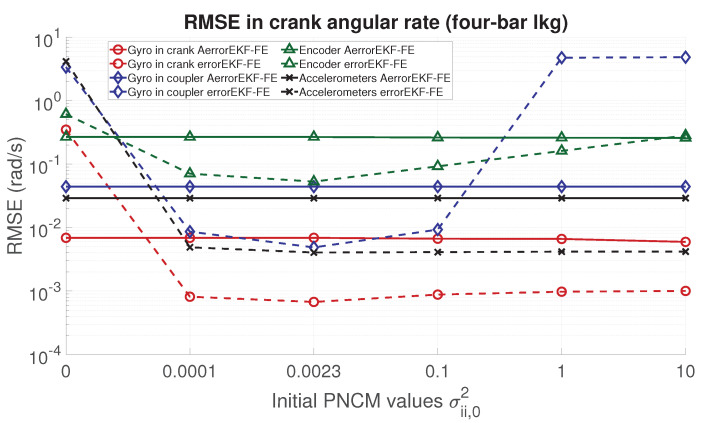
RMSE in the crank angular rate (velocity) provided by the observers for the four-bar linkage.

**Figure 8:**
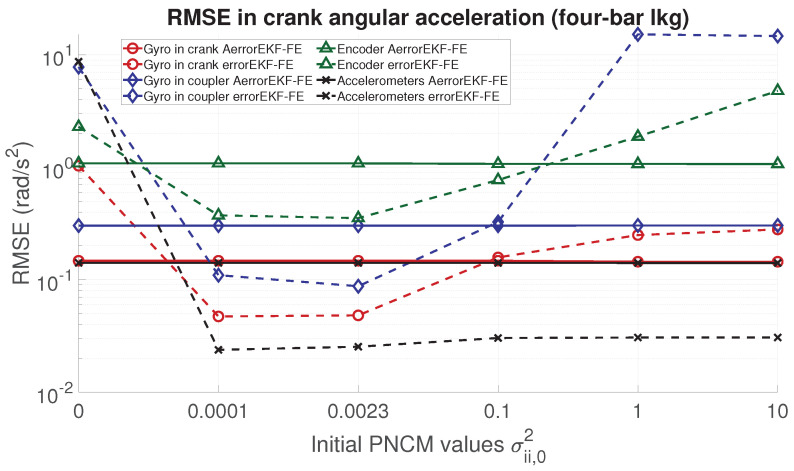
RMSE in the crank angular acceleration provided by the observers for the four-bar linkage.

**Figure 9:**
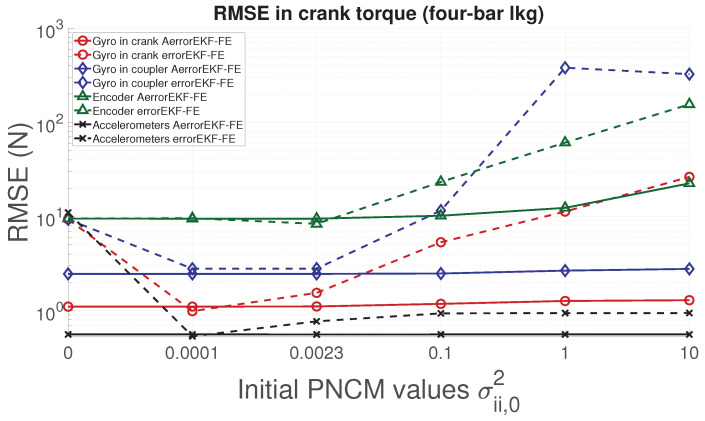
RMSE in the forces acting on the crank provided by the observers for the four-bar linkage.

In order to analyze the performance of the proposed adaptive filter, the RMSE for the position, velocity, acceleration and force acting in the degree of freedom of each mechanism is evaluated. From Figure 6, Figure 7, Figure 8 and Figure 9, the RMSE between the AerrorEKF-FE and errorEKF-FE are compared for the four-bar mechanism. Results show that the adaptive version of the errorEKF-FE leads in most of the test to a lower error in all the measured magnitudes. In fact, in some configurations, the RMSE is almost constant. This behavior is what could be initially expected: despite the different initial PNCM, the AerrorEKF-FE is capable of finding an optimal covariance that minimizes the errors in all tests.

**Figure 10:**
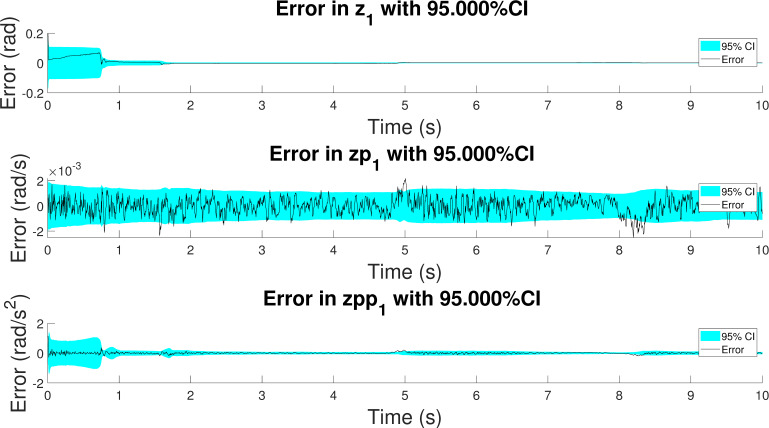
Error and confidence interval of the position, velocity and acceleration of the crank angle in the configuration which considers a gyroscope on the coupler.

The confidence intervals of the error in position, velocity and acceleration are shown in Figure 10 for one of the simulations. It can be seen how the errors converge to the true value and that the confidence intervals are consistent with the evolution of the error. Considering that the confidence interval is of 95%, it can be asserted that only the 5% of the estimations will have an error higher than 1.96 times the standard deviation of the variable (i.e., the square root of the diagonal elements of the covariance matrix, P, of Equation (21)). These plots also provide useful information about the observability of the system. Since the confidence interval is based on the PNCM elements, if the system were not observable, it could be expected an unlimited growth of the covariance of the states.

**Figure 11:**
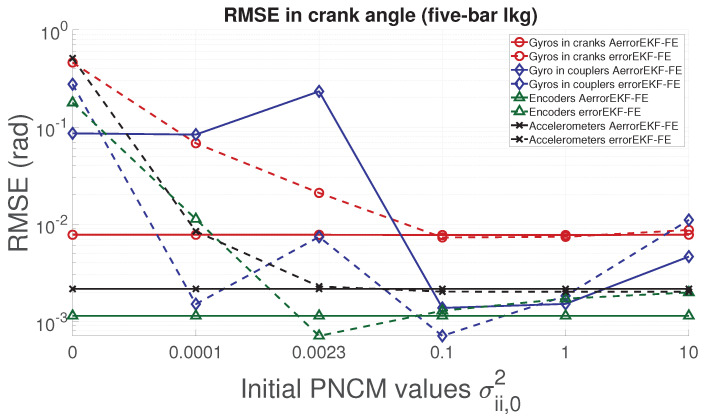
RMSE of the norm of the two crank angles (position) provided by the observers for the five-bar linkage.

**Figure 12:**
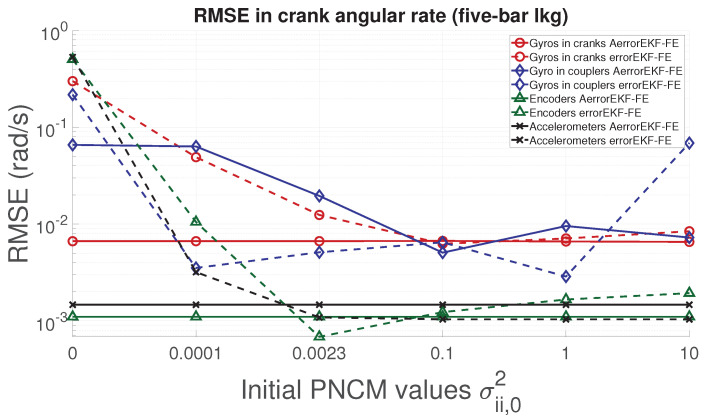
RMSE of the norm of the two crank angular rates (velocity) provided by the observers for the five-bar linkage.

**Figure 13:**
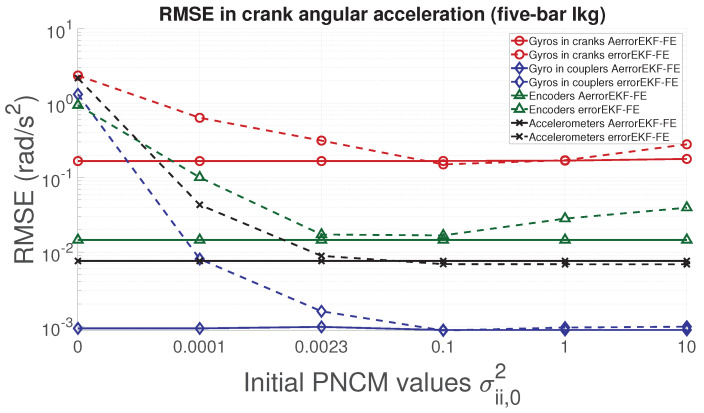
RMSE of the norm of the two crank angular accelerations provided by the observers for the five-bar linkage.

**Figure 14:**
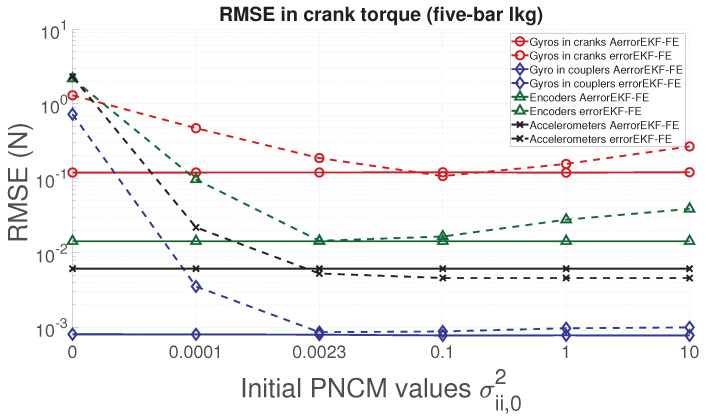
RMSE of the norm of the forces acting on the two cranks provided by the observers for the five-bar linkage.

With respect to the five-bar linkage, a similar trend can be appreciated from Figure 11, Figure 12, Figure 13 and Figure 14. Except for the crank angle and angular rate when using gyroscopes in couplers, the rest of the experiments show that the AerrorEFK-FE converges to accurate solutions in every tested case. Thus, it is able to estimate the value of the PNCM which minimizes the error.

### 3.2. Robustness Test

For evaluating the robustness of the filter, the four-bar linkage is equipped with a torsional spring actuating over the crank angle. In order to replicate a failure, at 5 s, the spring breaks. In this new scenario, the *real mechanism* continues the maneuver without the spring, while the *observer* is still considering its existence for the dynamics. This test is useful to analyze the response of the filter to unexpected changes. The *observer* is also under the errors of previous tests: $1\;m/s^{2}$ in gravity acceleration. In addition, the initial value of the crank angle has an offset of π/16rad.

**Figure 15:**
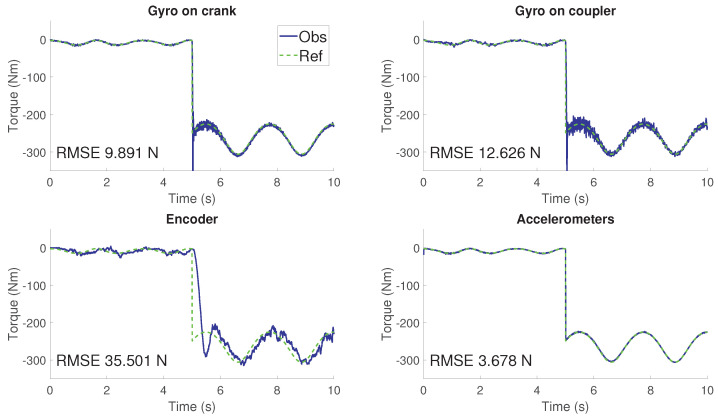
Torque estimation for the four-bar linkage for the spring failure scenario. *Obs* refers to the estimated torque and *Ref* refers to the theoretical value.

The results in terms of force estimation are shown in Figure 15. The estimated force can be understood as the torque, which would have to be applied to the crank to compensate the errors. It is equivalent to the difference between the force applied in the *real mechanism* and the *model*. It can be seen how, at the time of 5 s, the *observer* has to subtract the force in the crank. Since there is no spring in the *real mechanism*, the *observer* needs to apply a torque equal in magnitude but different in sign to the torque applied by its spring.

It can be seen how, except for the encoder configuration, the *observer* tracks the reference values. In addition, the filter responses with immediacy to the spring failure. This time response can be achieved by adjusting the innovation window length, seeking for a compromise between accuracy and time response. For this case, the window length is of 50 samples. Regarding the encoder configuration, the behavior is similar to what could be appreciated in [9]. There is a delay in the estimation and the noise is noticeable. It can be explained by the fact that obtaining acceleration from position information.

**Figure 16:**
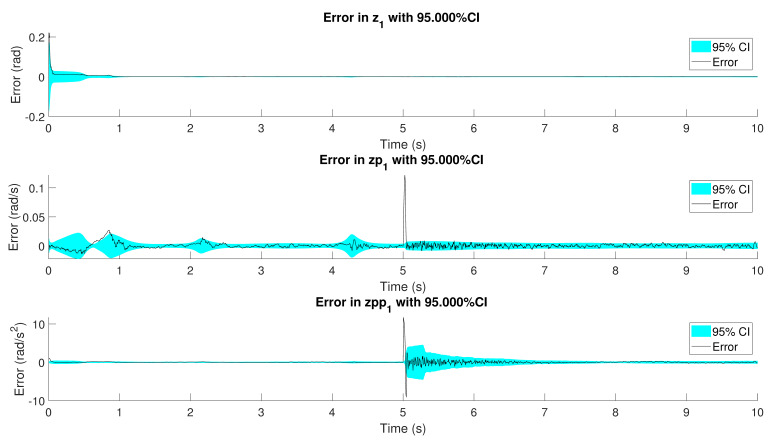
Error and confidence interval of the position, velocity and acceleration of the crank angle in the configuration which considers a gyroscope on the coupler.

With respect to the other three configurations, the estimations present a reduced noise, being the use of accelerometers the most accurate solution. The use of gyroscopes shows a good behavior before the spring breaks. Once this event takes place, both tests show a remarkable overshooting which is quickly corrected at expenses of higher noise. However, in both cases, it can be appreciated how the noise is being reduced with time. This is in line with what is shown in Figure 16.

It can be seen how the confidence interval becomes wider when the spring breaks. In terms of the PNCM, it means that the AerrorEKF-FE has detected the change in the *real mechanism*. To address this new modeling error, the filter increases the values of the PNCM giving more relevance to the sensor measurements. Once that the *observer* tracks the new scenario of the *real mechanism*, the covariances are reduced together with the noise of the estimations.

### 3.3. Computational Cost

For most of the industrial applications, it is required to achieve real-time performance. In previous works, the errorEKF-FE has proven to be capable of running in real time with complex multibody models [10]. Hence, it is of interest to analyze the increment in computational cost derived by the presented adaptive procedure.

It is important to remark that this work is developed in MATLAB${}^{®}$, which is not oriented to real-time applications. Hence, measuring the computation time of the algorithm is not a fair test. However, since the errorEKF-FE and AerrorEKF-FE are executed under the same conditions, a reference in the increment in computational cost can be derived. It can be seen in Table 5. From the results, it can be concluded that the AerrorEKF requires about the double of time than the error errorEKF-FE for computing the same simulation.

## 4. Discussion

From the presented results, it can be seen that the adaptive version of the errorEKF-FE solves one of the main drawbacks of the filter: setting the values of the process noise covariance matrix. The tests performed during this work show that, with independence of the initial assumptions on the PNCM, the AerrorEKF-FE converge to an accurate solution. As shown during the accuracy test, the AerrorEKF-FE is able to achieve a similar level of error in the estimations despite of the initial values of the PNCM. This allows to reduce the development time of multibody-based Kalman filters, where the determination of the statistics of the system is a tedious process. In addition, even though the maximum likelihood method depends on a sliding window of the past innovation, the filter has shown an acceptable response to sudden changes in the system. Through the simulation of a sudden failure in the *real mechanism*, the robustness of the filter is tested. The results show that the estimator is able to track the new situation and correct the new error with accuracy, without compromising the convergence of the simulation.

It can also be concluded that, although the measurement noise covariance matrix can also be estimated through the ML method, in this particular case it led to a filter divergence in the absence of accelerometers. When the estimator detects an error, it starts to rely on the corrections in the measurements instead of the model. However, through sensors in position or velocity, the acceleration cannot be obtained with accuracy and hence, the error cannot be corrected. This, together with the non-linearities of the model, leads to a divergence of the filter. In addition, the estimation of the MNCM did not offer an improvement of the estimations. However, as opposite from the PNCM, it is possible to obtain an acceptable initial value for the MNCM after characterizing the sensors or through the information provided by the manufacturer of each sensor.

The length of the sliding window for the innovation sequence is also an important factor and one of the main limitations of the approach. A large window size can result into wrong estimations in maneuvers with high variability. This is one of the known disadvantages of the ML method, since there does not exist a general rule in order to select the most suitable innovation window length. Algorithms such as Sage-Husa are focused on minimizing the impact of the window length by giving different weights to the elements of past events, giving higher weights to the more recent events.

Regarding the computational cost, the AerrorEKF-FE has lower efficiency than the errorEKF-FE due to the adaptive procedure. Estimating the process noise covariance matrix each time step entails an increment of the computations per time step, which is turned into a noticeable increment of computational cost. This limitation can be critical in real-time applications. It is necessary to test the computational cost of the method in each particular application. The efficiency of the estimator not only depends on the algorithm, but on the platform where it is going to be executed. This also implies to explore the possibility of increasing the efficiency of the solution. Since the estimation of the noise covariance matrices is independent from the state estimation each time step, the code execution can be parallelized, increasing the algorithm efficiency and reducing the computational cost of the approach.

## 5. Conclusions

This work presents an adaptive Kalman filter for state and input estimation based on multibody models (AerrorEKF-FE). The aim is to accurately estimate the noise covariance matrix of the estimator, which has shown to be a critical factor for accurate and robust observer performance. The method is tested on two different planar mechanisms: a four-bar linkage and a five-bar linkage. In addition, four different sensor configurations are considered, increasing the general application of the proposed solution. Several tests are presented in order to analyze the behavior of the filter in terms of accuracy and robustness.

The AerrorEKF-FE combines an error-state Kalman filter with an adaptive method based on the maximum likelihood criteria. This adaptive technique, commonly used for navigation estimation, has several assumptions which are not fulfilled in multibody dynamics. Even though adaptive algorithms have the capacity of overcoming some of the main difficulties of multibody based Kalman filtering, its application has never been explored. In this work, the selected adaptive method is adjusted to fit with multibody particularities, such as time-variant transition and observation matrices. Furthermore, the estimated matrix should be adapted to the shape of the covariance matrix (if known), increasing the accuracy of the observer.

The first test evaluates the accuracy of the filter under different initial values of the noise covariance matrix. The results are compared against the estimations obtained with a non-adaptive version of the filter (errorEKF-FE). The results show an improvement in accuracy with the use of adaptive techniques. In addition, it can be seen how the adaptive method allows to achieve similar results in terms of accuracy in spite of the initial assumption of the covariance noises.

In a second test, the robustness of the filter is studied through a simulation in which an unexpected event changes the system. The filter shows a quick reaction and corrects with accuracy the new error, without loosing the stability of the estimator.

The tests executed in this work and their results show the potential of adaptive Kalman filtering for multibody based estimation. Determining the noise covariance matrix (specially the noise of the process) in multibody based estimators is an actual limitation for the general use of this techniques in multibody applications. Through adaptive estimation, the uncertainties on the process noise covariance matrix can be solved and the robustness and accuracy of the estimators can be increased and guaranteed during different scenarios. However, this method also implies a reduction of the computational efficiency that should be considered in real-time applications.

In future works, the proposed adaptive filter should be applied in systems of more complexity, such as vehicle dynamics, where the state and input estimation is of high interest. In addition, techniques for reducing the associated increment in computational cost can be studied.

## Figures and Tables

**Table 1:** Properties of the four-bar linkage.

	Crank	Coupler	Rocker	Ground Element
Mass (kg)	2	8	5	-
Length (m)	2	8	5	10

**Table 2:** Properties of the five-bar linkage.

	Left Crank	Left Coupler	Right Coupler	Right Crank	Ground Element
Mass (kg)	3	1	2	3	-
Length (m)	0.5	2.062	3.202	0.5	3

**Table 3:** Characteristics of the sensors considered in this work.

	Encoder	Gyroscope	Accelerometers
Standard deviation	1.745 × 10${}^{-2}$ rad	9.839 × 10${}^{-4}$ rad/s	5.638 × 10${}^{-2}$ m/s${}^{2}$
Sampling frequency (Hz)	200	200	200

**Table 4:** Initial values of the PNCM.

Test	1	2	3	4	5	6
$\sigma_{0}^{2}$	0	0.0001	0.0023	0.1	1	10

**Table 5:** Computational cost analysis of the AerrorEKF-FE. The simulations tested correspond to the use of position sensors.

	Simulated Time (s)	Computing Time (s)	
		errorEKF	AerrorEKF
Four-bar linkage	10	1.5931	3.1955
Five-bar linkage	10	2.2551	4.7234

## Data Availability

Not applicable.
